# Recapping the Features of SARS-CoV-2 and Its Main Variants: Status and Future Paths

**DOI:** 10.3390/jpm12060995

**Published:** 2022-06-18

**Authors:** Miguel A. Ortega, Cielo García-Montero, Oscar Fraile-Martinez, Paolo Colet, Ardak Baizhaxynova, Kymbat Mukhtarova, Melchor Alvarez-Mon, Kaznagul Kanatova, Angel Asúnsolo, Antonio Sarría-Santamera

**Affiliations:** 1 Department of Medicine and Medical Specialities, Faculty of Medicine and Health Sciences, University of Alcalá, 28801 Alcala de Henares, Spain; miguel.angel.ortega92@gmail.com (M.A.O.); cielo.gmontero@gmail.com (C.G.-M.); oscarfra.7@hotmail.com (O.F.-M.); mademons@gmail.com (M.A.-M.); 2 Ramón y Cajal Institute of Sanitary Research (IRYCIS), 28034 Madrid, Spain; 3 Department of Medicine, Nazarbayev University School of Medicine, Nur-Sultan 010000, Kazakhstan; paolo.colet@nu.edu.kz (P.C.); ardak.baizhaxynova@nu.edu (A.B.); kymbat.mukhtarova@nu.edu.kz (K.M.); kaznagul.kanatova@nu.edu.kz (K.K.); 4 Immune System Diseases-Rheumatology, Oncology Service an Internal Medicine (CIBEREHD), University Hospital Príncipe de Asturias, 28806 Alcala de Henares, Spain; 5 Department of Surgery, Medical and Social Sciences, Faculty of Medicine and Health Sciences, University of Alcalá, 28801 Alcala de Henares, Spain

**Keywords:** SARS-CoV-2, variants, COVID-19, VOC, VOI, de-escalation, epidemiology

## Abstract

Over the two years that we have been experiencing the Severe Acute Respiratory Syndrome Coronavirus-2 (SARS-CoV-2) pandemic, our challenges have been the race to develop vaccines and the difficulties in fighting against new variants due to the rapid ability of the virus to evolve. In this sense, different organizations have identified and classified the different variants that have been emerging, distinguishing between variants of concern (VOC), variants of interest (VOI), or variants under monitoring (VUM). The following review aims to describe the latest updates focusing on VOC and already de-escalated variants, as well as to describe the impact these have had on the global situation. Understanding the intrinsic properties of SARS-CoV-2 and its interaction with the immune system and vaccination is essential to make out the underlying mechanisms that have led to the appearance of these variants, helping to determine the next steps for better public management of this pandemic.

## 1. Introduction

Coronaviruses are infectious agents that cause a wide variety of diseases in animals and humans, where they are endemic and responsible for up to 15 to 30% of annual respiratory tract infections [1]. Despite this fact, coronaviruses were not considered to be especially pathogenic for humans until the irruption of the Severe Acute Respiratory Syndrome Coronavirus (SARS-CoV), first recognized in China in November 2002 [2]. Since the onset of SARS-CoV, experts warned about the potential of coronaviruses to emerge and evolve causing human and veterinary outbreaks, owing to their ability to recombine, mutate, and infect multiple species and cell types [1,3]. These properties may imply that immune cells have not recognized novel emerging coronaviruses, which are also able to hijack and evade the host immune response [4]. Thus, ten years later, in 2012, a novel type of coronavirus, Middle East respiratory syndrome coronavirus (MERS-CoV), appeared in Middle Eastern countries [5]. The last member of currently recognized pathogenic human coronaviruses is represented by the SARS-Coronavirus 2 (SARS-CoV-2), first identified in late December 2019 in Wuhan, China and causative of Coronavirus Disease-19 (COVID-19) [6]. SARS-CoV, MERS-CoV, and SARS-CoV-2 have been identified as potential epidemiological threats. However, notwithstanding that the fatality rate of SARS-CoV-2 seems to be notably inferior to the rates caused by SARS-CoV and especially by MERS-CoV, the total number of individuals infected by SARS-CoV-2 has been vastly greater, leading to an unprecedented situation [7]. Indeed, since the World Health Organization (WHO) declared the COVID-19 a pandemic situation two years ago (11 March 2020), the number of registered cases has already reached 516 million cases, leaving 6.24 million deaths in its wake, in a manner that has fiercely impacted healthcare systems. Not only has normalcy changed, but also the research work: epidemiologic data, prevention and containment measures, urgent vaccination plans, and the search for antivirals to treat the disease have been prioritized.

The continuous evolution of the infection in different hosts across the globe and the consequent new emerging variants have also been under surveillance throughout this time. In this highly dynamic context, we aim to focus on the state of the art regarding SARS-CoV-2 variants, where we will only focus on those that recently have been categorized as “variants of concern” (VOC), as they have proven to have some detrimental effects and negative implications for this long pandemic. Initially, some critical information will be summarized regarding the molecular biology, virology, and immunology of SARS-CoV-2, in order to understand the emergence and impact of the different variants, aiding to create a general perspective of the current and possible scenarios of the greatest infectious pandemic of the 21st century so far. Understanding the co-evolution of SARS-CoV-2 with the immune system is key in order to address the appearance of new variants and their clinical management.

## 2. A General Perspective for SARS-CoV-2

### 2.1. Molecular Biology and Taxonomy

SARS-CoV-2, the etiological agent of COVID-19, belongs to the *Betacoronavirus* genus, the same as MERS-CoV and SARS-CoV according to clustering based on genetic similarities [8]. These three viruses emerged in the present century, switching from animal hosts to the human species, with the last of them, SARS-CoV-2, spreading at pandemic level [9]. The term “*Coronavirus*” was coined due to the crown shape that their proteins imitate when observed by electron microscopy. In the Baltimore classification of viruses, based on their manner of synthesizing mRNA, SARS-CoV-2 belongs to group IV. This means that a positive-sense single stranded RNA (+ssRNA) can be translated directly into proteins and, once entered into the cell, it can use host machinery to replicate additional viruses. In this Baltimore division group, there are several classes of virus with envelope or not [10]. The one that concerns us presents envelope and belongs to the *Nidovirales* order and *Coronaviridae* suborder, whose members represent the largest-known RNA viruses, with genomes ranging 25–32 kb and virions with diameters of 118–140 nm. Following current taxonomy, SARS-CoV-2 is finally classified into the *Coronavirinae* family, *Orthocoronavirinae* subfamily, and *Betacoronavirus* genus.

The non-segmented +ssRNA genome presents a structure of mRNA with a 5′ cap and a 3′ poly-A tail of approximately 29.7 kb in size [11]. The complete genome sequence of SARS-CoV-2 has been provided in *GenBank* (accession no. MN908947.3) since January 2020 [12,13]. The genome structure starts with a 5′ untranslated region (UTR), followed by an open reading frame (ORF) 1ab encoding non-structural accessory proteins, then gene S for spike glycoprotein, ORF3, genes E and M encoding envelope and membrane elements, respectively, ORFs 7, 8 and 10, gene N for nucleocapsid, and finally 3′UTR [14]. The sequence results in 5′-replicase-S-E-M-N-3′.

The spike protein contains two subunits S1 and S2. S1 contains a receptor-binding domain (RBD) that recognizes cellular host receptor angiotensin-converting enzyme 2 (ACE2), and S2 arbitrates viral envelope fusion to cell membrane by a six-helical bundle [15]. Then, SARS-CoV-2 relies on RNA-dependent RNA polymerase (RdRp) for replication and transcription. It is proposed that the subjacent mechanisms of RdRp dimer dissociation and template-switching could be behind the production of subgenomic RNAs [16]. Comprehension of these mechanisms has even been hypothesized as its ‘Achilles heel’ for antiviral drugs design [17,18].

Although human *Betacoronaviruses* mainly infect the respiratory tract with mild symptoms, the more severe SARS-CoV, MERS-CoV, and SARS-CoV-2 emerged over the last two decades and also affect pneumocytes and upper respiratory tract cells, compromising life in some cases [19]. The problem that has concerned us today for two years lies in the high contagion and mutation capacity of novel SARS-CoV-2, which has resulted in more pathologically aggressive and life-threatening strains for some groups of population and age. More precisely, Wang et al. [20] defined the mutation rate of SARS-CoV-2 as 6.677 × 10^−4^ substitutions per site per year, and the nucleotide mutation rate of the S gene as 8.066 × 10^−4^ substitutions per site per year, which is at a medium level compared with other RNA viruses. Phan warned in his work in July 2020 about the presence of a wide range of mutations and deletions either in coding or in non-coding regions in 86 genomes of SARS-CoV-2, affirming its mutagenic capacity and rapid evolution [21]. A possible reason is the dissociation of polymerase complex, now that these viruses present a high rate of RNA recombination during replication [19,22]. Nevertheless, some next-generation sequencing (NGS) approaches have analyzed the evolution of lineages in this short period of time, identifying an increasing number of mutations and variability in S and N proteins, but more conserved sequences in RdRp [23]. Importantly, these mutations can lead to an enhanced replication and transmissibility of the virus, and in turn this can favor further mutations, thus explaining the success and rapid evolution of SARS-CoV-2 [24].

### 2.2. Infection Cycle

First, the spread route is through droplets containing viral particles produced when a person sneezes or coughs. These droplets preferably enter new hosts via nostrils, although it can be through eyes or mouth [25]. After being transmitted by exposure to infectious respiratory fluids, the SARS-CoV-2 cycle starts with the recognition of the large S glycoprotein that extends from the envelope, by the site of RBD in S1. The principal specific binding to the cellular receptor ACE2 allows membrane fusion through S2 and, consequently, endocytosis [26]. However, there is broad entry tropism of SARS-CoV-2, given that other cellular proteins are a target for RBD: transmembrane serine protease 2 (TMPRSS2), kidney injury molecule-1 (Kim-1), and neuropilin-1 (NRP-1). Thus, characteristically, it affects multiple cell types and organs (respiratory, urinary, digestive, cardiovascular, etc.) [27]. Moreover, previous studies in SARS-CoV showed that ACE2 and TMPRSS2 receptors are co-expressed in type II pneumocytes, demonstrating them to be the main viral target cells [28].

The RNA viral genome is released into a host cell, whose machinery is hacked for translation of ORF1a and ORF1b to synthesize polyproteins pp1a and pp1b, respectively, which results in 16 nonstructural proteins (nsps) post-translationally by proteolytic cleavage. Of these, 15 nsp conform to the viral replication and transcriptional complex (RTC) that contains enzymes for RNA processing, modification, and exonuclease with proofreading function. [19]. Of note is nsp5 (Mpro, 3CLpro), with protease function, which has been a therapeutic target focus due to its conserved role and is key for viral cycle [29]. Then, replication starts with −ssRNA synthesis as a template for +ssRNA synthesis, from which translation proceeds to generate nsp and RTC. Next, formation of viral structural proteins N harbor +ssRNA, resulting in the assembly of new virions; then, S, E, and M are inserted via endoplasmic reticulum and Golgi bodies, forming the viral envelope for the matured virus, which hence can be ejected from the cell by exocytosis [30,31].

**Figure 1:**
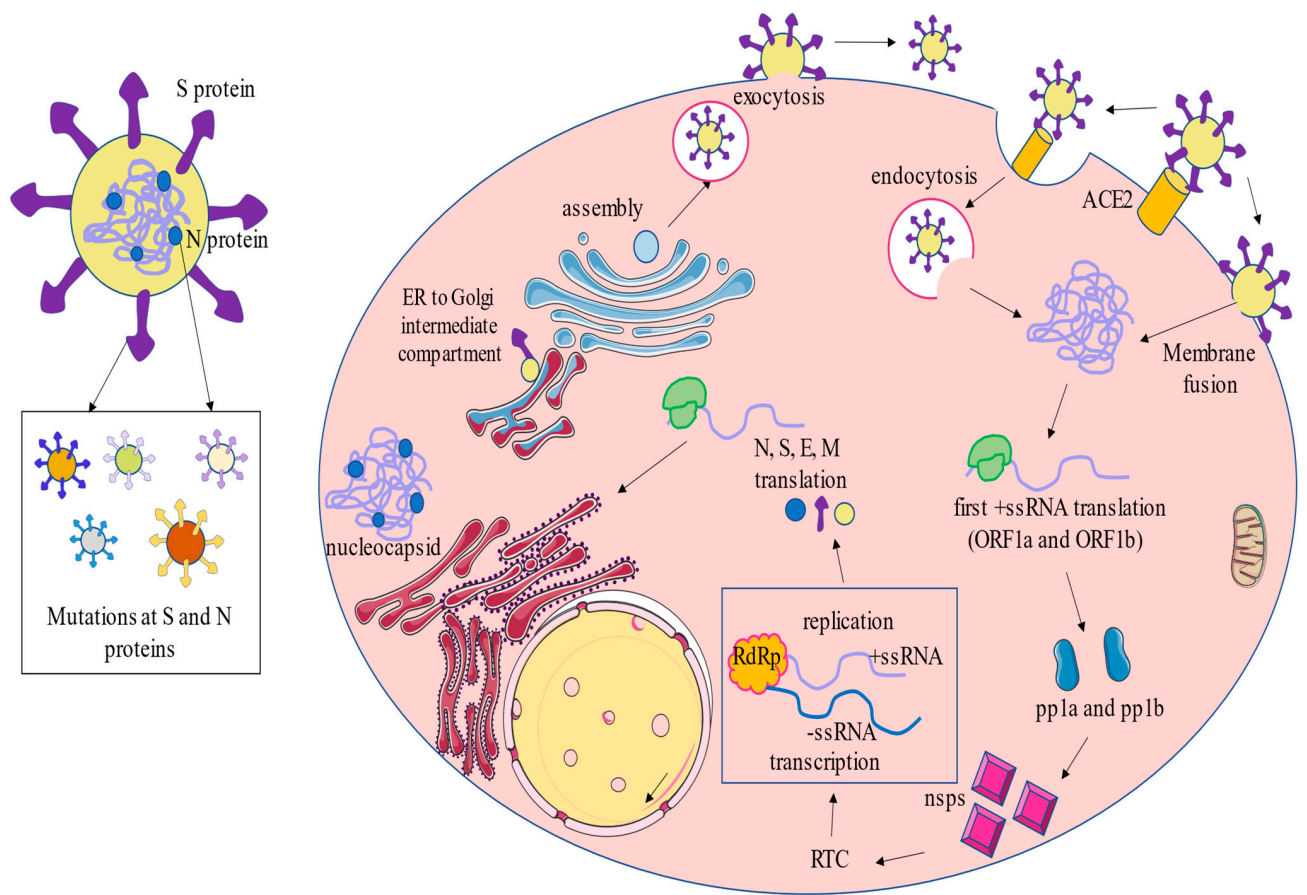
A global picture of SARS-CoV-2 structure and viral cycle. As shown, SARS-CoV-2 is mainly composed by the spike (S), nucleocapsid (N), envelope (E), and membrane (M) proteins, and a +ssRNA. SARS-CoV-2 enters into the cell through the binding of the S protein with ACE2, leading to the formation of endosomes or through membrane fusion. Then, the ssRNA is translated, starting with the open reading frame (ORF) 1a and ORF1b regions codifying polyproteins (pp)1a and pp1b. Through proteolytic cleavage, these polyproteins form 16 nonstructural proteins (nsps), which are related to the replication and transcriptional complex (RTC). In this sense, of note is the role of nsp12, also known as RNA-dependent RNA polymerase (RdRp), in the replication and transcription of the ssRNA. Then, the above-mentioned viral S, N, E, and M proteins are translated and packaged in the endomembranous system, from the endoplasmic reticulum (ER) to the Golgi complex, where it is finally assembled, and a set of viral particles are finally released through exocytosis. Last but not least, it should be highlighted that both S and N proteins can easily mutate during the viral replication cycle. As will be later discussed, this property of the SARS-CoV-2 is directly related to the onset and development of novel variants.

Structural characteristics and viral cycle are summarized in Figure 1.

### 2.3. Immune Response and Vaccinology

An individual’s favorable response to SARS-CoV-2 depends, firstly, on the proper function of immune system. Particularities of COVID-19 immunopathogenesis may depend on different host factors like age, gender, ABO blood group, and multiple risk factors [32]. In fact, the pathogenicity of this kind of Coronavirus exerts severe inflammatory responses, especially in people with previous comorbidities or in the elderly, although there are some patients that may even respond asymptomatically. It is difficult to define the proportion of asymptomatic people infected by SARS-CoV-2. However, a systematic review and meta-analysis conducted by Chen et al. [33] including 130,123 infections from 241 studies in different regions and countries worldwide found approximately 31,411 asymptomatic individuals. In other words, they concluded that ~1 in 5 infections by SARS-CoV-2 were asymptomatic in 2020. Regarding symptomatic patients, the vast majority appears to present mild or moderate COVID-19 presentation. In a study conducted by Wu & McGoogan [34] in 72,314 patients, 81% had mild COVID-19 (including patients without pneumonia or with mild pneumonia), whereas 14% were severe and 5% critical. Vulnerable groups may end up suffering a cytokine storm with accompanying systemic hyperinflammation and respiratory insufficiency (acute respiratory distress syndrome, ARDS), thromboembolic events, or death [35,36]. These aspects, as well as lymphopenia and eosinopenia, are rarely manifested in other respiratory viral infections [37]. This uncontrolled pro-inflammatory response is highly represented by TNF-α, IL-1, IL-6, IL-12, and IFN-γ, and entails a cascade of events with multi-organ damage promoted by inflammatory monocyte recruitment in alveoli [38,39,40]. Some of these cytokines have been evaluated as prognostic factors for patients with severe COVID, demonstrating their efficacy, especially in the case of IL-6, IL-10, and the granulocyte colony-stimulating factor (GCSF) [39]. Lymphopenia explains the insufficient antiviral T-cell response combined with innate immune response disorder [14,36]. Importantly, lymphopenia and a low lymphocyte/leukocyte ratio has been related to an augmented risk of intensive care unit (ICU) admission or death by COVID-19 [41].

After infection, three main stages can be distinguished in COVID-19, as Siddiqi & Mehra described in their work [36]. In the first stage, there are only mild symptoms (fever, dry cough) leading to innate (TLR-3,7,8 activation) and adaptative immune responses (IgM and IgG against N and S). When virus is not eliminated at this level, there is a second stage with pneumonia with or without hypoxia, and aberrant cytokine expression starts. If immune response is still not favorable to SARS-CoV-2 elimination, the hyperinflammation status is triggered, in what is called the third stage (ARDS and multi-organ failure) [35].

Humoral response is varied in the population. Seroprevalence studies indicate many infected people develop anti-S and anti-N antibodies, but titers change differently across time. It seems that in either vaccination or infection, both kind of antibodies are produced, but only anti-S (S-IgA and S-IgG) are maintained, and anti-N soon disappear (N-IgA and N-IgG) [42,43]. In this sense, a remanent question has been whether testing for antibodies provides useful information or not. Beyond clinical trials and research utility, clinical value is still limited even though public interest is high [44].

On the other hand, quick vaccine development and optimal vaccine programs worldwide have been crucial to attenuate dissemination and adverse immune response in different populations [45], although so-called herd immunity seems out of reach now that protection is imperfect, especially in asymptomatic infection. In these cases, propagation is unavoidable, and the continuous capacity to mutate makes the virus able to evade vaccine-elicited immunity [46]. Nevertheless, vaccination continues to strengthen our damaged sense of normalcy, as it can save the lives of those who would not otherwise be prepared to face a different and unpredictable version of coronavirus. To date, the WHO recognizes 10 vaccines approved worldwide, divided into categories of (1) mRNA-based, which encompass Pfizer/BioNTech (BNT162b2) and Moderna (mRNA-1273-); (2) inactivated virus, encompassing Bharat Biotech (COVAXIN), Sinopharm (Covilo/BBIBP-CorV), Sinovac (CoronaVac); (3) non-replicating viral vectors, encompassing Janssen (Johnson & Johnson-Ad26.COV.2S), Oxford/AstraZeneca (AZD1222), and Covishield (Oxford/AstraZeneca formulation), and (4) protein subunits, encompassing Novavax (NVX-CoV2373) and Covovax (Novavax formulation). The European Medicines Agency (EMA) only approves for use BNT162b2, mRNA-1273, NVX-CoV2373, Ad26.COV.2S, and AZD1222, whereas BNT162b2, mRNA-1273, and Ad26.COV.2S are the only vaccines approved by the U.S. Food & Drug Administration (FDA) [47].

Regarding its immunological role, COVID-19 vaccines elicit strong T-cell responses, which may provide protection even without seroconversion, helping to explain the relevance of vaccination [48]. Likewise, it must also be considered that natural SARS-CoV-2 infection boosted with vaccination can provide greater long-term immunization than only two doses of vaccination [49,50], with this protection being directly correlated with the severity of COVID-19 [51]. Thus, a third dose of vaccination has been shown to provide further protection in patients not naturally infected by SARS-CoV-2 [52]. As will be subsequently discussed, there are different SARS-CoV-2 variants which can evade vaccine-induced immunity. This could be due to the above-mentioned mutations occurring specially in the S and N proteins, affecting their transmission, virulence, and antigenicity [53]. Regarding these novel variants, while antibody breadth against viral variants is lower after infection in comparison to vaccination, it seems to improve over several months, also generating more specific antibodies against variant antigens [54]. Furthermore, the positive aspects of hybrid immunity have been discussed. This concept refers to the combined effects of infection with different SARS-CoV-2 variants in the same individual, with evidence related to improvements in B-cell stimulation and antibody production [55]. However, other authors refer to the hybrid concept as the effect of natural infection by any variant combined with later vaccines doses or vice versa (which seems to be the preferred definition), while claiming that this form of hybridity confers the greatest protection against SARS-CoV-2 [56].

## 3. Main SARS-CoV-2 Variants

As previously described, the proper molecular biology and characteristics of SARS-CoV-2 have led to the evolution and emergence of novel variants since the discovery of this novel coronavirus in December 2019. In this section, we will focus on the most prominent features and differences of the main SARS-CoV-2 variants, the mutations that have occurred, epidemiological data, immune interaction, and other possible evidence with consequences for vaccination. The rapid evolution has shown that many ramifications can arise from the same lineage; for example, the Delta variant has produced approximately 200 subvariants, and Omicron has 4 prominent subvariants with great spreading ability. As previously mentioned, most common mutations that can be found are within S and N proteins [23].

In general terms, the epidemiological classification of the variants follows the SARS-CoV-2 Interagency Group (SIG), identifying variants of concern (VOC), variants of interest (VOI), variants under monitoring (VUM), and variants of high consequences (VOHC). Currently, there are neither VOHC nor VOI; only VOC and VUM are considered. Moreover, it is noteworthy that some of these variants have changed in their assignment in the classification given the different ability of each of them to spread. In this sense, there are other classifications, such as those given by the European Center for Disease Prevention and Control (ECDC)—which considered in July 2021 a new category designated as de-escalated variants [57]. The variants included in this category were either those that currently are not circulating or those that, despite having been circulating for a long time, did not show any impact on the epidemiological situation and are not associated with any concerning properties. The different included agents and the rationale for their de-escalation are summarized in the official website of ECDC [58].

It is of note that the classification of some variants may be different depending on the institutional considerations as time goes on. As of 5 June 2022, the ECDC only considers as VUM the variants BA.3 and BA.2 + L452X, which are being observed and studied; to date, more evidence is required before drawing any conclusion for these variants. There are a number of de-escalated variants without any impact on the actual situation, such as Epsilon, Lambda, Iota or Theta. Beta, Delta, Gamma, and Omicron are at this time considered VOCs by this institution; nevertheless, the WHO, which also considered the Alpha variant until recently, states that only Delta and Omicron are currently VOCs [59]. However, and as will be later discussed, there are some particular lineages derived from the initial designated variants; the epidemiological surveillance conducted in this field is quite tight.

### 3.1. Alpha

The Alpha (B.1.1.7) variant was first discovered in United Kingdom from a sample obtained in September 2020 [60]. Alpha was the dominant variant in the United Kingdom from December 2020 to May 2021, when it was substituted by Delta until December 2021, when it was surpassed by Omicron [61]. By March 16, more than 275000 cases of this variant had been reported in this country, which was followed by USA, Germany, and other European countries [62]. Thus, this variant has infected an important percentage of the global population, although it is now considered a de-escalated variant due to its low representation in the wake of Delta and Omicron. At least 22 mutations have been recognized in this variant, including a N501Y mutation of the spike protein, which increases the affinity of RBD with ACE2; the P681H mutation, associated with an improved entry into the cell; and the D614G mutation in the spike protein, which also enhances infectivity [63]. All these mutations resulted in an improved transmissibility of this variant over others, with an estimated 50% to 100% higher reproduction number [64].

In respect to the clinical presentation in hospitalized patients, differences between the Alpha variant and the wild-type SARS-CoV-2 were virtually non-existent [65,66], although there were some studies claiming an increased mortality and severity in patients with this variant [67]. Moreover, the evidence seems to indicate that the efficacy of vaccination regarding the transmissibility of the Alpha variant is higher than the Delta, and two vaccination doses can exert significant protective effects against this variant [68,69,70]. It seems that the protection against this variant is quite similar and shows notable effectiveness against different types of vaccines, including mRNA-based, inactivated virus, viral vectors, and protein subunits [45,71,72,73], although some studies have found a slight difference between them. For instance, among mRNA-based vaccines, mRNA-1273 appears to display greater efficacy than BNT162b2 [74]. On the other hand, it is also important to consider the immune response generated by the individual, as there is some evidence suggesting that subjects partially or fully vaccinated with lower virus-neutralizing antibody levels were more likely to be infected by the Alpha variant [75]. The detection of IgA and IgG in the nasopharyngeal fluid appears to be directly correlated with an improved response against the Alpha variant, supporting the relevance of the humoral response to control this infection [76]. Furthermore, some of the mutations present in the Alpha variant outside the spike protein appear to modulate the innate immune system (i.e., N protein, Orf9b, and Orf6), therefore facilitating immune evasion [77]. In this sense, despite this variant having little presence today, these results illustrate the need to consider the efficacy of the vaccines against specific variants and explore further vaccination strategies and additional antigenic targets in next-generation SARS-CoV-2 vaccines [78].

### 3.2. Beta

The Beta (B.1.351) variant emerged in South Africa in October 2020, being the most representative variant in this country until the onset of Omicron, and extended to 115 countries [79]. This variant presents nine mutations in the spike protein (L18F, D80A, D215G, R246I, K417N, E484K, N501Y, D614G, A701V). Three of them, K417N, E484K, and N501Y, have been associated with a raised infectivity and mortality in comparison to the Alpha variant, which only possesses N501Y mutation [80,81].

In terms of the clinical implications of this variant, an increased risk of hospitalization, ICU admission, and mortality has been observed in comparison to Alpha and Gamma, being similar to Delta variant, although less severe in comparison [82,83]. It has been demonstrated that the mutations that occurred in the S protein lead to an abnormal recognition by the immune system of the Beta variant [84]. Fortunately, the T-cell immunity is preserved despite this fact, and natural immunity seems to offer an adequate protection against reinfection with the Beta variant even 1 year after the primary infection [85]. On the other hand, the antibody responses against this variant show a substantial reduction in RBD binding and neutralization [86]. In this sense, two doses of vaccination are critical to acquire high levels of neutralizing antibodies and high antibody titers. Vaccination has also shown a reduced effectivity against this variant, especially regarding its transmission and mild-to-moderate presentations, although it exerts a protective role against severe or fatal disease [87]. Some studies have found that the efficacy of vaccines may be similar to that obtained with Alpha. For instance, whereas BNT162b2 confers a protection of 95% against Alpha, the estimated effectiveness against the Beta variant was 94% [88,89]. Besides, the vaccination programs followed in some countries when this variant was circulating almost a year ago did not significantly affect control over the pandemic [90]. Conversely, other vaccines seemed to display different results regarding their efficacy towards Beta when compared to Alpha. For example, Novavax presented 86% efficacy against the Alpha variant, whereas a 60% efficacy was reported with Beta [91]. Currently, similar to Alpha and Gamma variants, the dissemination of this strain has slowed, being now considered a de-escalated variant.

### 3.3. Gamma

Gamma (P.1) variant was detected in Japan in December 2020, although it first emerged in Brazil, evolving from a local B.1.1.28 clade in late November 2020 and replacing the parental lineage in less than 2 months, especially during the second wave [92]. Gamma variants present at least 23 characteristic mutations, and similar to Beta and Gamma variants, also present K417N/T, E484K, and N501Y mutations, which are responsible for higher infectivity [81]. While this variant has also extended to other countries, especially those in South and Center America, the Gamma variant has become endemic in Brazil, especially in the State of Amazonas, where different Gamma Plus variants have been evolving. These variants harbor additional S protein mutations, making them more transmissible than the parental P.1 [93,94,95].

Evidence has failed to find any significant association between Gamma variant infections and severity of COVID-19 symptoms. However, its rapid dissemination and socioeconomic and public health limitations could explain the augmented number of cases and deaths during the second wave in Brazil [96]. Likewise, as both Beta and Gamma variants carry identical RBD changes, they display significant reductions in neutralization by convalescent plasma, although this reduction was greater in Beta than in Gamma variant [97]. Less information is available in terms of the vaccination success against the Gamma variant. However, it seems that in general terms, mRNA-based vaccines, AZD1222, and CoronaVac are effective in preventing symptomatic COVID-19 and severe infections against Alpha, Beta, Gamma or Delta variants [98].

### 3.4. Delta

SARS-CoV-2 Delta variant (B.1.617.2) was first reported in India on December 2020, competing with the pre-existing B.1.617.1 (Kappa) and B.1.1.7 (Alpha) variants [99]. Epidemiologically, the Delta variant played a central role in the second wave, presenting an increase of 108% in hospitalization risk, 235% in ICU admission rate, and 133% in chance of death than the original variant [100]. These data could be due to the higher viral load of the Delta variant in comparison to other variants, being more transmissible among humans (especially through aerosols), without directly affecting mortality as such [101,102]. Because of both of these factors, the use of FFP2 respirators when indoors has been proposed as one of the most effective preventive measures against Delta and Omicron variants [103]. However, some recent data have failed to find an increased viral load from the Delta variant [104], concluding that the increased infectivity of this variant may be caused by other factors. Currently, this variant is thought to be the most prevalent in many countries, such as the USA. This variant presents 10 main mutations in the spike protein: T19R, (G142D*), 156del, 157del, R158G, L452R, T478K, D614G, P681R, D950N [105,106]. Overall, the mutations presented by Delta and the previous variants (Alpha, Beta, and Gamma) show an enhanced adaptation to human ACE2, being also able to broaden their host ranges in comparison to the original SARS-CoV-2 [107].

Importantly, different Delta SARS-CoV-2 subtypes such as Delta Plus have been emerging, with unique mutation profiles that are nevertheless highly correlated, which may be motivated by the need to preserve the structural integrity of the virus. Because of this, not only the Delta variant, but also Delta Plus and other subtypes are currently being collectively monitored and surveilled by experts [108,109]. Thus, although we refer to “delta variant”, these different viruses are also included herein.

Regarding clinical manifestations, the Delta variant appears to present a lessened time interval between disease onset to hospitalization in comparison to the wild-type variant; it also shows significant changes in its hematological profile. Moreover, there has been higher hospital admission or emergency care attendance risk for patients with COVID-19 infected with the Delta variant compared to those affected with the Alpha variant, as well as an increased transmissibility [110,111]. Fortunately, early diagnosis and a full vaccination routine could protect patients from severe disease progression [112,113]. In vitro, B.1.617.2 has been shown to be 6-fold less sensitive to serum neutralizing antibodies from recovered individuals, and 8-fold less sensitive to vaccine-elicited antibodies, compared with wild-type SARS-CoV-2. In addition, this variant has demonstrated higher replication efficiency, an upgraded entry in the cells due to S protein mutations, and poorer serum-neutralizing titers in AZD122 vaccinees than in BNT162b2 vacinees [114]. Indeed, despite the similar efficacy of two doses of AZD122 and mRNA-based vaccines (91% versus 92%), the efficacy of AZD122 against severe COVID-19 declined over time to 69% by 20 weeks from second dose. The efficacy of mRNA vaccines declined in the first 10 weeks from second dose, but more slowly thereafter to 93% (88–96) at 20 weeks from second dose [115]. Furthermore, the evidence seems to indicate that mRNA-based vaccines are more effective against this variant in comparison to non-mRNA vaccines, such as the above mentioned AZD122 or Ad26.COV.2S vaccines [116,117,118].

Overall, these facts and cumulative evidence explain the rapid dissemination and permanence of this variant, which currently remains to be the case.

### 3.5. Omicron

The Omicron variant (B.1.1.529) was first identified in Botswana in November 2021, and it was first reported to the WHO on November 24, upon which it was classified as VOC two days later [119]. This variant harbors more than 30 mutations in the spike protein with 15 of them affecting the RBD, which is key for the interaction with ACE-2 [120]. These mutations have enabled a very rapid dissemination of this variant, affecting more than 2000 people in 57 countries in a short period of two weeks. By the end of 2021, 89 countries reported the presence of Omicron in their genome sequences [121,122]. The origin of this virus is quite involved. There are some hypotheses suggesting that the emergence of this variant could have arisen in an immunosuppressed individual with the Human Immunodeficiency Virus (HIV) or through a recombination with the human common cold coronavirus, although there are other studies hypothesizing a possible animal (mouse) origin of this variant [123,124]. Other plausible explanations consider the possible circulation and evolution of the variant in a remote population under intense evolutionary pressure, low vaccine coverage, or inequity in vaccination, which permitted an ideal evolutionary context for Omicron [125].

Regarding its clinical manifestations, it seems that Omicron is associated with a mild presentation compared to Delta. However, Omicron has been eclipsing Delta variant in multiple countries. It is difficult to establish the intrinsic severity of this variant, as in comparison to Delta, the global percentage of vaccinated people may explain some of these differences, other factors notwithstanding [126]. However, there are some worrying facts behind what makes Omicron a VOC. For instance, it is known that vaccinated patients and convalescent individuals previously infected with other variants present a noteworthy reduction of sera neutralization titers against Omicron. Fortunately, patients with the third vaccine dose or those previously infected by the Delta variant may show an enhanced antibody response against Omicron [127,128]. Indeed, while the cellular response induced by two vaccination doses or infection was not affected, the third vaccination dose provides greater protection against symptomatic or non-symptomatic infections, transmission, and serious manifestations [129]. Interestingly, patients infected with Omicron have an improved immune response that not only neutralized this variant, but also others, such as Delta, thereby gradually reducing the prevalence of this variant [130]. To date, the evidence seems to support that a mRNA-vaccine booster, especially the mRNA-1273 vaccine, offers the greatest protection against Omicron, and heterologous booster vaccination regimens did not appear to be inferior to the homologous booster vaccination regimens [131]. However, it is also true that while this third dose significantly improves the immune response against Omicron, the immunization is incomplete. Thus, some studies are starting to evidence the benefits of a fourth vaccine dose of either BNT162b2 (Pfizer–BioNTech) or mRNA-1273 (Moderna) administered 4 months after the third dose. In this sense, a fourth dose of mRNA vaccine appears to be immunogenic and safe, although to what extent a fourth dose may be effective is still controversial [132,133,134,135]. In this context, experts hold that there is an urgent need for updated vaccines that may aid in controlling the spread and dissemination of this variant, especially for single-dose regimes [136].

Finally, there has been some controversy regarding Omicron’s role in the pandemic. On the one hand, as Omicron causes less-severe COVID-19, it may well be a contributor to ending the pandemic [137]. Conversely, despite this tendency, it is also likely that more variants will continue to appear, as humans and SARS-CoV-2 strains are meant to coexist. Indeed, as of 5 June 2022, there are specific Omicron sublineages apart from B.1.1.529 that have been appearing and should be mentioned here. These are the cases of BA.1, BA.1.1, BA.2, BA.3, BA.4, and BA.5, and the hybrid Omicron variants XD, XE, and XF.

### 3.6. Omicron Sublineages

In the preceding months to this review, a set of Omicron sublineages have been detected; BA.1 and BA.1.1 were the first to be reported and presented a rapid dissemination [138]. In a short period of time, two additional variants, BA.2 and BA.3, were also noticed. Of these, BA.2 shared 32 mutations with BA.1, presenting 28 distinct mutations, whereas BA.3 mutations were nearly all in common with BA.1 and BA.2, except for one [139]. Currently, BA.1 and BA.2 are considered VOCs, whereas BA.3 and a specific BA.2+, L452X, are VUM [58]. However, despite BA.1 and BA.1.1 being the predominant variants initially, BA.2 is currently replacing these sublineages and becoming the dominant variant [140]. Regarding the initial evidence of vaccination and immune evasion of BA.1 and BA.2 sublineages, it seems that vaccine boosting regimens, especially via the third dose, may provide sufficient protection against Omicron-induced disease, even if they may evade polyclonal-neutralizing antibody responses [141]. Indeed, it is likely that there are not many differences regarding immune evasion between BA.1 and BA.2, but the last presents an enhanced transmissibility [142]. In addition, some authors are starting to demonstrate that these different sublineages may present different virological features; further studies are warranted to deepen understanding of the pathological mechanisms of Omicron variants [143].

Finally, the BA.4 and BA.5 sublineages have been recently identified, and the ECDC now considers them VOCs [58]. Currently, the prevalence of these variants is growing in South Africa, which is being closely surveilled by experts, although preliminary data have not indicated any notable change in the rates of COVID-19 cases and hospitalizations [144]. However, more evidence is needed before drawing any conclusion on these specific sublineages.

### 3.7. Omicron-Hybrid Variants

There are some specific hybrid sublineages designed as XD, XE, and XF, although little evidence is currently available. XD and XF are a combination of Delta and BA.1, whereas XE is a combination of BA.1 and BA.2. The XD variant has a BA.1 S gene incorporated into a Delta genome, and because of that, it has been popularly designated as “Deltacron”; it is present in several different European countries [145]. Conversely, XF has not been detected since 15 February, in the UK. The XE genome is mostly represented by BA.2, including the S gene, and probably presents a greater growth rate, although more date is required [146]. The XD sublineage is currently considered a VUM by the ECD and the WHO, although the XE sublineage has not been considered by these institutions yet. Future studies will elucidate the precise role of these sublineages in the worldwide pandemic.

## 4. Conclusions and Future Directions

**Figure 2:**
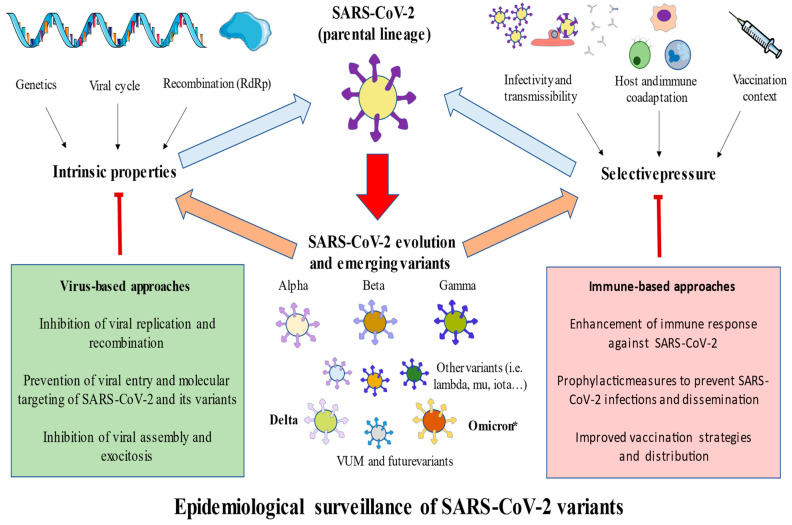
A general picture of the evolution and emergence of SARS-CoV-2 variants. As shown, due to the intrinsic properties of the virus (genetics, recombination, and viral cycle) and selective pressure (host and immune coadaptation, infectivity and transmissibility efficacy, and the actual vaccination context), a set of variants has been developing with enhanced features compared to their parental lineages, including greater transmission, infection rates, or circulation. Of them, currently Delta and Omicron are considered VOCs, where Omicron * represents the dominant variant worldwide. Simultaneously, there may be novel variants appearing, as well as further lineages of previously established variants. To limit the impact and slow the continuous evolution of SARS-CoV-2, virus- and immune-based approaches and, more prominently, prophylactic measures and improved vaccination strategies are needed.

Since the onset of SARS-CoV-2 pandemic two years ago, there has been a wide number of people affected worldwide, with important implications not only for the infected and healthcare professionals, but also for all of society, including in terms of economical and mental health concerns [147,148]. The emergence of novel variants presents an important challenge in putting an end to this long pandemic. As summarized in Figure 2, the intrinsic properties of SARS-CoV-2, including its recombination and genetic basis and the selective pressure exerted on it, favors the evolution of SARS-CoV-2 parental lineages, leading to the appearance of different variants. Overall, multiple variants have been identified, although the Alpha, Beta, Gamma, Delta and Omicron variants, as well as their lineages, have been recognized as VOCs due to their prominent effects in the pandemic. In Table 1, we summarize some of the most important knowledge derived from these variants. In order to limit the effects of these variants or the evolution of SARS-CoV-2, different strategies can be discussed here. These approaches may be either directed to the virus or the immune system. For instance, the inhibition of the viral cycle and its replication may be an interesting method to limit the evolution and infection of SARS-CoV-2, and the enhancement of the immune system can also benefit the antiviral response against the virus [106]. However, these approaches have their limitations, as there are asymptomatic individuals in which the evolution of SARS-CoV-2 can occur. Thus, the most effective strategies are the following: (1) prophylactic measures, including the use of masks and social distance specially when indoors; (2) improved vaccination strategies, emphasizing the need for ensuring the global distribution of vaccines, especially in low- and middle-income countries; (3) study the number of doses and type of vaccines with the greatest efficacy. Moreover, while the administration of fourth-dose vaccine is starting to provide some evidence on benefit, there is a critical need for the development of novel vaccines against SARS-CoV-2, especially single doses against the Omicron variant.

## Figures and Tables

**Table 1:** A summary of the main SARS-CoV-2 variants.

Variant	Current Epidemiological Classification (ECDC)	First Report	Mutations in the Spike Protein of Interest	Clinical Manifestations	Immune Evasion	Vaccination Cocerns	References
Alpha (B.1.1.7)	De-escalated variant	United Kingdom, September 2020.	N501Y, D614G, P681H	Similar presentation to wild-type SARS-CoV-2, although some studies observed an increased mortality and severity	Possible influence in the innate immune system due to some mutations in the N protein, Orf9b, and Orf6	Two doses of vaccine, especially mRNA-1273, appears to exert protective effects against this variant.	[8,9,45,61,62,63,64,65,66,67,68,69,70,71,72,73,74,75,76,77,78]
Beta (B.1.351)	De-escalated variant	South Africa, May 2020.	K417N, E484K, N501Y, D614G, A701V	Increased risk of hospitalization, ICU admission, and mortality in comparison to Alpha and Gamma variants, but less than Delta	Spike mutations of Beta variant are not recognized by T-cells, but the immunity is equally preserved even 1 year after primary infection. E484K mutation may lead to escape from immune response	Some vaccines have presented a reduced efficacy against this variant regarding its transmission and mild-to-moderate presentations, although they exert a protective role against severe or fatal disease	[8,9,80,91]
Gamma (P.1)	De-escalated variant	Brazil, November 2020.	K417T, E484K, N501Y, D614G, H655Y	No significant association between Gamma variant infections and the severity of COVID-19 symptoms has been reported	E484K mutation may lead to escape from immune response	Little information available, but it seems that mRNA-based vaccines, AZD1222, and CoronaVac are effective in preventing symptomatic COVID-19 and severe infections against this variant	[8,9,81,92,93,94,95,96,97,98]
Delta (B.1.617.2)	Variant of concern (VOC)	India, October 2020.	L452R, T478K, D614G, P681R.	Delta variant presents a lessened time interval between disease onset to hospitalization in comparison to the wild-type variant, while also showing significant changes in hematological profile	6-fold less sensitive to serum neutralizing antibodies from recovered individuals, and 8-fold less sensitive to vaccine-elicited antibodies, compared with wild-type SARS-CoV-2.	While the overall efficacy of the vaccines are diminished with this variant, mRNA-based vaccines (Pfizer and Moderna) provide greater protection against this variant	[8,9,100,101,102,103,104,105,106,107,108,109,110,111,112,113,114,115,116,117,118]
Omicron (B.1.1.529)	Variant of concern (VOC)	Identified in multiple regions in November 2021.	A67V, del69-70, T95I, del142-144, Y145D, del211, L212I, ins214EPE, G339D, S371L, S373P, S375F, K417N, N440K, G446S, S477N, T478K, E484A, Q493R, G496S, Q498R, N501Y, Y505H, T547K, D614G, H655Y, N679K, P681H, N764K, D796Y, N856K, Q954H, N969K, L981F	Despite some evidence suggesting that this variant leads to milder clinical presentations than Delta, it is difficult to establish the intrinsic severity of this variant due to different factors (i.e., the global percentage of vaccinated people)	Mutations in the ACE-2 binding site boost the immune escape of this variant, especially from neutralizing antibody responses	Three vaccination doses are needed to elicit a more appropriate immune response against symptomatic or non-symptomatic infections, transmission, and serious manifestations. Being infected by Omicron seems to confer a greater protection against this and other variants.	[8,9,119,120,121,122,123,124,125,126,127,128,129,130,131,132,133,134,135,136,137]
Omicron sublineage BA.1 and BA.1.1	Variant of concern (VOC)	First recognized in South Africa and Botswana in November 2021; they were predominant variants until the onset of BA.2	A67V, del69-70, T95I, G142D, del143-145, N211I, del212, ins215EPE, G339D, S371L, S373P, S375F, K417N, N440K, G446S, S477N, T478K, E484A, Q493R, G496S, Q498R, N501Y, Y505H, T547K, D614G, H655Y, N679K, P681H, N764K, D796Y, N856K, Q954H, N969K, L981F BA.1.1 = BA.1 with the spike R346K substitution	Similar to B.1.1.529	Boosted immune escape, similar to BA.2	Similar to B.1.1.529	[138,139,140,141,142,143]
BA.2	Variant of concern (VOC)	Dominant Omicron variant	G142D, N211I, del212, V213G, G339D, S371F, S373P, S375F, T376A, D405N, R408S, K417N, N440K, S477N, T478K, E484A, Q493R, Q498R, N501Y, Y505H, D614G, H655Y, N679K, P681H, N764K, D796Y, Q954H, N969K	Similar to B.1.1.529	Boosted immune escape, similar to BA.1, but increased transmissibility.	Similar to B.1.1.529	[138,139,140,141,142,143]

## Data Availability

Not applicable.
